# Caspase inhibition impaired the neural stem/progenitor cell response after cortical ischemia in mice

**DOI:** 10.18632/oncotarget.6803

**Published:** 2015-12-30

**Authors:** Ahmed M. Osman, Susanne Neumann, H. Georg Kuhn, Klas Blomgren

**Affiliations:** ^1^ Karolinska Institute, Department of Women's and Children's Health, Karolinska University Hospital, Stockholm, Sweden; ^2^ Center for Brain Repair and Rehabilitation, Institute of Neuroscience and Physiology, University of Gothenburg, Gothenburg, Sweden

**Keywords:** stroke, neurogenesis, migration, Q-VD-OPh, neuroinflammation

## Abstract

Cortical ischemia induces proliferation of neural stem/progenitor cells (NSPCs) in the subventricular zone (SVZ) and provokes migration of these cells toward the injured area. Despite sustained migration of NSPCs for an extended period of time after injury, they do not appear to survive. Here, we hypothesized that the anti-apoptotic broad-spectrum caspase inhibitor Q-VD-OPh would increase NSPC survival in the injured cortex. However, contrary to our expectations, caspase inhibition did not promote NSPC survival and cortical neurogenesis. On the contrary, it abolished ischemia-induced proliferation and decreased the number of migrating neuroblasts in the injured cortex. Moreover, caspase inhibition decreased the levels of the chemoattractant chemokine CCL2 (MCP-1) and the pro-inflammatory cytokine IL-1β. We hence for the first time show that caspase inhibition abrogates the response of NSPCs to an ischemic injury, presumably by inhibiting the production of pro-inflammatory factors. Thus, caution is warranted if anti-apoptotic strategies are applied for neuroprotection.

## INTRODUCTION

Neural stem/progenitor cells (NSPCs) residing in the subventricular zone (SVZ) respond to brain injuries. After stroke, NSPC proliferation increases and a subset divert from their regular migratory path, the rostral migratory stream (RMS) to the olfactory bulb [1], and migrate toward the injury site [2, 3]. The NSPCs persist migrating to the peri-infarct area for at least one year after striatal [4] and cortical ischemia [5]. However, despite the continuous migration, generation of new striatal neurons is rare, and cortical neurogenesis is not detectable in mice [2, 5]. In humans, generation of new cortical neurons after ischemia is also absent [6] although the presence of immature neurons in the ischemic penumbra was evident [7]. It has been suggested that the newborn NSPCs fail to reach maturity due to the non-permissive, inflammatory microenvironment in the injury site, and that NSPCs therefore undergo apoptosis [8, 9].

In this study, we hypothesize that inhibition of caspase-mediated apoptosis using the pan-caspase inhibitor Q-VD-OPh (QuinolineValAsp(Ome)-CH2-O-Phenoxy) might be of value and would promote NSPC survival in the ischemic cortex. Q-VD-OPh is a broad-spectrum caspase inhibitors that has greater potency and specificity in blocking caspases and apoptosis [10, 11]. Compared to the widely used pan-caspase inhibitor Z-VAD-FMK (Z-Val-Ala-Asp(Ome)-CH2 F), the carbobenzoxy blocking group (Z) is replaced with a quinolone derivative (Q), the tripepeptide sequence VAD is reduced to VD, and the toxic fluoromethyl ketone (FMK) replaced with the nontoxic difluorophenoxy (OPh) group. The aspartic acid derivative in Q-VD-OPh forms an irreversible thioester bond with the active site cysteine of the caspase with subsequent displacement of the OPh group [10]. Advantages include that Q-VD-OPh crosses the blood brain barrier [11], and the low toxicity, enables long-term systemic administration.

Here, we induced cortical ischemia in mice using a photothrombotic stroke model followed by systemic treatment with Q-VD-OPh. Cortical neurogenesis and NSPC migration were analyzed, postulating that apoptosis inhibition would increase the number of surviving NSPCs in and around the ischemic lesion.

## RESULTS AND DISCUSSION

**Figure 1:**
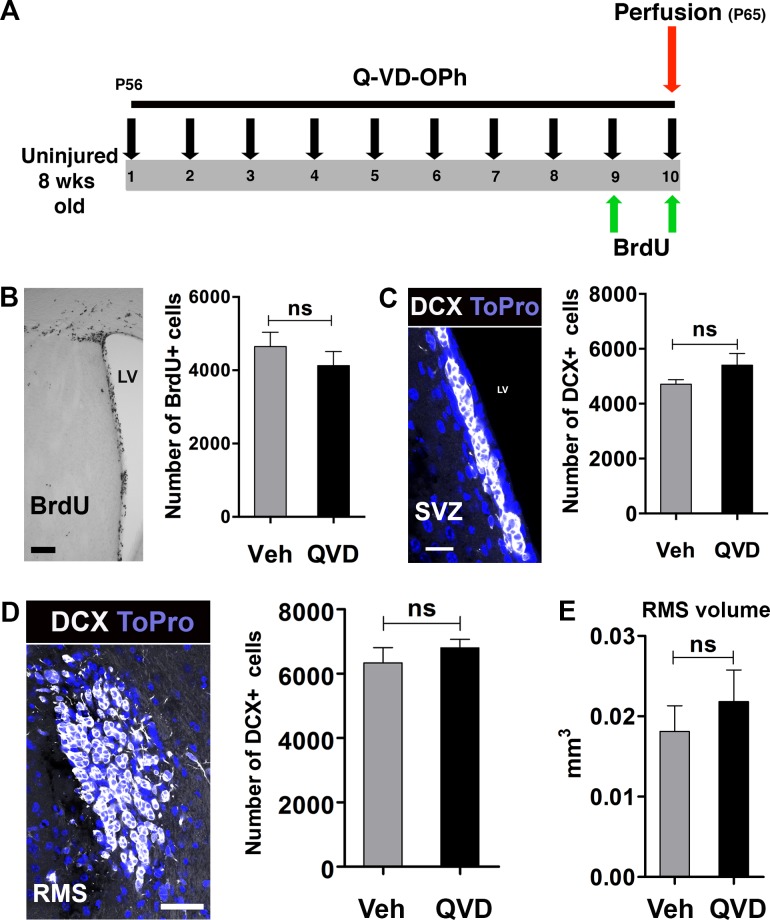
The effect of Q-VD-OPh on neural stem/progenitor cells in the intact, uninjured brain **A.** The experimental design with 10 days of Q-VD-OPh (QVD; *n* = 9) or vehicle (Veh; *n* = 8) administration. P = Postnatal day **B.** Analysis of proliferation in the SVZ. The image shows BrdU staining in the SVZ and the bar graph shows quantification data. **C.** Analysis of DCX+ cells in the SVZ. The confocal image displays DCX staining (white) in the SVZ. Blue is the nuclear staining ToPro. The bar graph shows quantification data. **D.** Quantification of DCX+ cells in the RMS. The confocal image represents DCX staining in the RMS in a coronal section, and the bar graph shows the quantification results. **E.** The volume occupied by DCX+ cells in the RMS. Data are presented as mean ± SEM. LV = lateral ventricle; ns = not significant. Scale bar = 100, 20 and 40 μm in **B.**, **C.** and **D.**, respectively.

Neural stem/progenitor cells (NSPCs) in the subventricular zone (SVZ) continuously divide and migrate to the olfactory bulb through the rostral migratory stream (RMS). In previous studies, deficiency of apoptosis-inducing factor, and knockout of the pro-apoptotic gene Bax were shown to reduce NSPC proliferation and migration [12, 13]. Therefore, we wanted to investigate if administration of Q-VD-OPh would affect these parameters under physiological conditions. Animals were treated with either Q-VD-OPh (QVD) or vehicle (Veh) for 10 days (Figure 1A). Proliferating cells were labeled with BrdU during the last 2 days of the treatment. Our results showed no difference in the number of BrdU+ cells between animals treated with Q-VD-OPh or vehicle (*P* = 0.36) (Figure 1B). When we analyzed the generation of neuroblasts in the SVZ and their migration in the RMS, no difference in the number of DCX+ cells between the treatment groups was observed, neither in the SVZ (*P* = 0.19) nor in the RMS (*P* = 0.43) (Figure 1C and 1D). Moreover, no difference in the volume of the RMS was detected, as judged by the volume occupied by DCX+ cells (*P* = 0.48) (Figure 1E). These data indicate that the anti-apoptotic Q-VD-OPh has no influence on NSPC proliferation or migration under physiological conditions.

**Figure 2:**
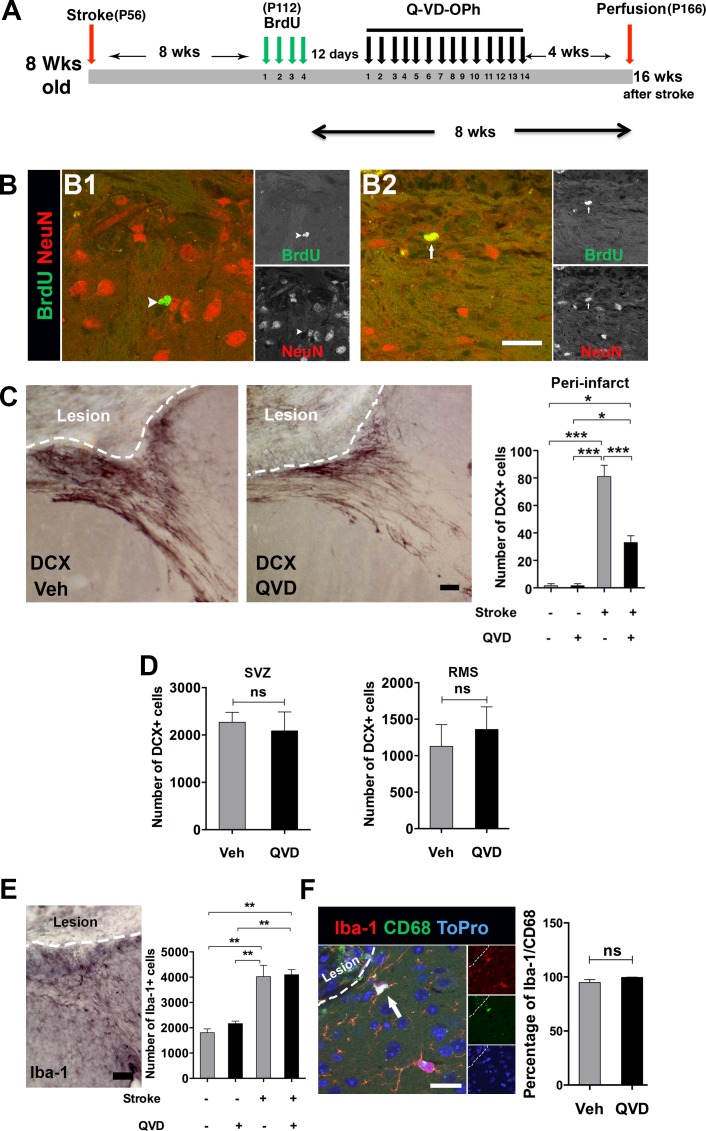
Assessment of cortical neurogenesis and migration after treatment with Q-VD-OPh late after stroke **A.** The experimental design where animals subjected to cortical ischemia and treated either with Q-VD-OPh (QVD) or vehicle (Veh). P = postnatal day **B.** Confocal images show BrdU (green) and NeuN (red) staining in the peri-infarct cortex. The arrowhead in **B1.** indicates a BrdU+ cell for which no colocalization with NeuN was detected, while the arrow in **B2.** points to one of the rare cells where colocalization was considered. **C** Analysis of DCX+ cells in the peri-infarct cortex 16 weeks after induction of ischemia. The images show DCX+ cells in the peri-infarct cortex of the treatment groups, and the bar graph shows quantification data. *P = 0.02, ***P < 0.0001. **D.** Quantification of DCX+ cells in the SVZ and the RMS in ischemic animals. **E.** Analysis of microglia in the peri-infarct cortex. The image represents Iba-1 staining in ischemic animal, and the bar graph shows the quantification data. **P < 0.001. **F.** A confocal image showing CD68 expression (green) in Iba-1+ cells (red). The nuclear staining ToPro is blue. The bar graph displays the percentage of Iba-1+ cells also expressing CD68. Data are presented as mean ± SEM. ns = not significant. Scale bar = 20 μm in **B.** and **F.**, and 50 μm in **C.** and **E.**. *n* = 4 for sham controls, and *n* = 8 for each of the ischemia groups (Q-VD-OPh and vehicle).

In a previous study [5], we could not detect generation of newborn cortical neurons after cortical stroke when we birth-dated NSPCs during the first ten days after ischemia, a period believed to be the peak of the inflammatory response after stroke [8]. Here, we hypothesized that NSPCs born at later time points after ischemia might have a better chance of surviving and possibly giving rise to mature cortical neurons. Cortical ischemia was induced in 8-week-old mice. We labeled the newborn NSPCs with BrdU 2 months after induction of ischemia. To promote survival of the newly generated neural progenitors, Q-VD-OPh or vehicle were administered starting 12 days after the last BrdU injection, a period just before neuroblasts convert to mature neurons, and maintained for 2 weeks, during the maturation process [14]. Animals were sacrificed 1 month after the termination of the treatment (Figure 2A). Very few newborn neurons (BrdU+/NeuN+) in the peri-infarct cortex were detected, only in 2 animals per treatment (Figure 2B), suggesting that even migrating progenitors born long after injury, beyond the inflammatory reaction, very seldom become mature cortical neurons, and that caspase inhibition did not promote additional cortical neurogenesis after ischemia.

As neural progenitors migrate to the ischemic cortex for an extended period of time [4, 5], we next intended to test whether administration of Q-VD-OPh would affect the migration of NSPCs to the injured cortex. As expected, cortical ischemia resulted in elevated numbers of DCX+ cells in the peri-infarct cortex compared with sham animals (Figure 2C). Unexpectedly, treatment with Q-VD-OPh resulted in 60% fewer migrating neuroblasts in the peri-infarct cortex in ischemic animals (Figure 2C). Therefore, we asked whether Q-VD-OPh treatment led to accumulation of neuroblasts in the SVZ due to impaired migration of DCX+ cells to the peri-infarct cortex or affected migration in the RMS after ischemia. We quantified the number of DCX+ cells in the SVZ and the RMS of the ischemic animals, but no differences were detected between the treatment groups, neither in the SVZ (*P* = 0.7) nor in the RMS (*P* = 0.6) (Figure 2D), indicating that Q-VD-OPh only influenced DCX+ cells migrating to the injury site. This suggests that cues attracting neuroblasts toward the injured cortex, and/or promoting proliferation of cells, are altered by Q-VD-OPh.

**Figure 3:**
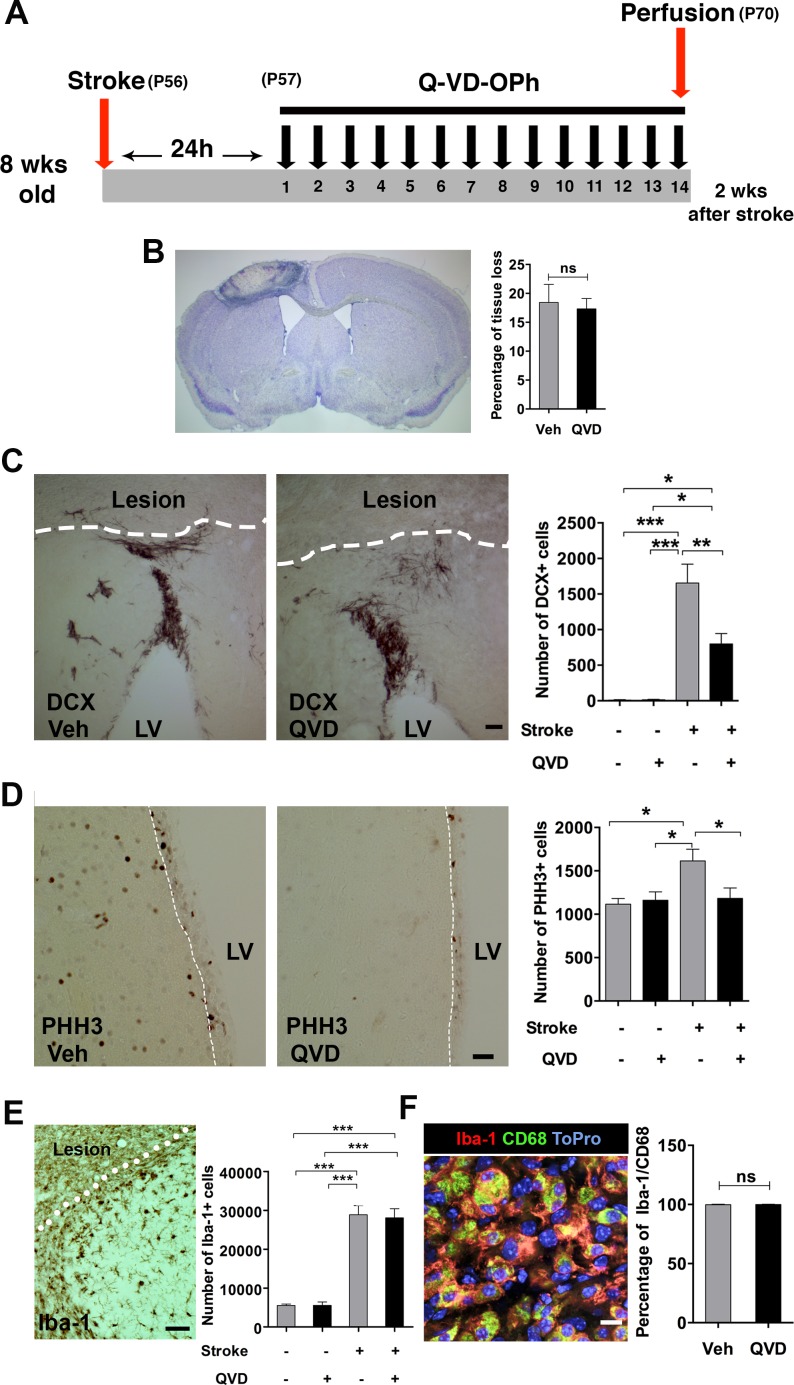
The short-term effect of Q-VD-OPh on the NSPC response after stroke **A.** The experimental design, where Q-VD-OPh (QVD) or vehicle were administered 24h after induction of stroke and continued for 2 weeks. P = Postnatal day **B.** Measurement of tissue loss after ischemia. Nissl staining was performed (the image) and the percentage of tissue loss in ischemic animals was calculated (bar graph). **C** Quantification of the DCX+ cells in the injured cortex after 2 weeks. The images show the DCX+ migrating cells in the peri-infarct cortex of the treatment groups, and the bar graph shows the quantification data. *P = 0.013, **P = 0.005, ***P < 0.0001. **D.** Analysis of proliferation in the SVZ at 2 weeks. The images represent PHH3 staining in the SVZ (indicated by a white dashed line) of ischemic animals from the two treatment groups, and the bar graph shows the quantification data. *P = 0.01. **E.** Analysis of Iba-1+ cells in the peri-infarct cortex at 2 weeks. ***P < 0.0001 **F.** Assessment of microglial activation in the peri-infarct cortex after 2 weeks. The confocal image shows CD68 expression (green) in Iba-1+ cells (red). Blue is the nuclear staining ToPro. The bar graph shows the percentage of coexpression of Iba-1 and CD68. Data are presented as mean ± SEM. LV = Lateral ventricle; ns = not significant. Scale bar = 100 μm in **C.**, 50 μm in **D.** and **E.**, and 10 μm in **F.**. *n* = 4 for sham controls, and *n* = 7 for each of the ischemia groups (Q-VD-OPh and vehicle).

Brain injury is associated with neuroinflammation, and pro-inflammatory cyto/chemokines are major stimulants for NSPC migration after ischemic injury [15, 16]. Thus, we studied the microglia/macrophage response in the peri-infarct cortex. Indeed, ischemia resulted in increased numbers of Iba-1+ cells in the ischemic animals compared to their sham counterparts. However, no difference was detectable between the treatment groups (Figure 2E), and virtually all microglia expressed the activation marker CD68 [17, 18] (Figure 2F). Of note, this analysis was done 4 weeks after termination of the treatment (16 weeks after the injury) (Figure 2A), and might not fully reflect the effects of Q-VD-OPh on microglia. To address this, we performed a third experiment where Q-VD-OPh was administered daily for 2 weeks starting 24 h after induction of ischemia, when cell death resulting from ischemia likely is completed and no impact of the inhibitor on infarct size is expected [19]. Brains were collected 2 h after the last injection for histology and protein analysis (Figure 3A). We confirmed that the percentage of tissue loss was unaffected by Q-VD-OPh treatment (*P* = 0.8) (Figure 3B). The number of neuroblasts in the peri-infarct cortex was 50% lower in ischemic animals receiving Q-VD-OPh (Figure 3C), confirming that injury-induced migration was equally inhibited at an earlier time point after ischemia. The number of proliferating NSPCs in the SVZ peaks after 1-2 weeks in response to brain ischemia, and returns to baseline approximately 6 weeks after stroke [2, 3, 9], therefore we next quantified the number of PHH3+ (proliferating) cells in the SVZ, and found that Q-VD-OPh treatment completely prevented the ischemia-induced increase in proliferation (Figure 3D). No difference in the numbers of Iba-1+ cells or the percentage of Iba-1+/CD68+ cells was detected in the peri-infarct cortex (Figure 3E and 3F, respectively). Together, these results indicate that the main effect of caspase inhibition was to prevent the injury-induced increased proliferation in the SVZ, subsequently resulting in decreased migration.

**Figure 4:**
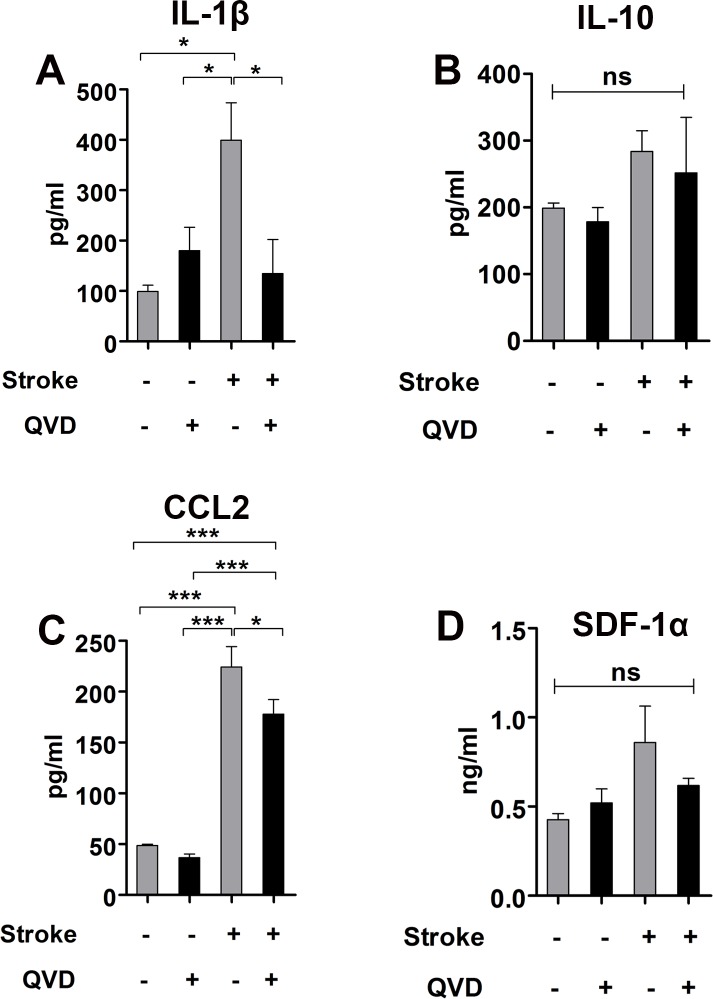
Cytokine analysis 2 weeks after stroke **A.** Analysis of IL-1β. *P < 0.05 **B.** Analysis of IL-10. **C.** Analysis of CCL2. *P < 0.05, ***P < 0.0001 **D.** Analysis of SDF-1α. ns = not significant. *n* = 4 for sham controls, and *n* = 5 for each of the ischemia groups (Q-VD-OPh and vehicle). Data are presented as mean ± SEM.

Activated microglia/macrophages can undergo pro-inflammatory (M1) or anti-inflammatory (M2) polarization and both express CD68 [17, 20]. We used ELISA to measure the levels of IL-1β and IL-10, cytokines associated with M1 and M2 microglia/macrophages, respectively. We found less IL-1β in ischemic animals treated with Q-VD-OPh, while the levels of IL-10 remained unaltered (Figure 4A and 4B), suggesting that Q-VD-OPh influenced the pro-inflammatory M1 polarized microglial/macrophages and the anti-inflammatory M2 remained unchanged, and accordingly, no difference in activation could be seen as judged by CD68 expression.

The chemokine (C-C motif) ligand 2 (CCL2) and the stromal cell-derived factor 1 α (SDF-1α) trigger recruitment of microglia/macrophages and neural progenitors to site of injury *via* their expression CCR2 and CXCR4, respectively [15, 16]. When we measured the levels of these chemokines, we found 25% reduction in CCL2 in ischemic animals treated with Q-VD-OPh (Figure 4C). SDF-1α was not significantly reduced by Q-VD-OPh (Figure 4D). These results suggest that Q-VD-OPh mainly interrupted CCL2-mediated injury-induced neural progenitor migration. Contrary to our findings, intracerebroventricular administration of a cocktail of the pan-caspase inhibitor Z-VAD-FMK together with caspase-3 and -9 inhibitors after cortico-striatal stroke increased neuroblasts migration to the damaged area in rats, and this was associated with increased levels of SDF-1α [9]. As mentioned in the introduction, Q-VD-OPh is a superior caspase inhibitor compared to Z-VAD-FMK [10, 11]. Additionally, we applied Q-VD-OPh intraperitoneally, and systemic administration enables caspase inhibition also in peripheral and infiltrating immune cells.

Overall, we showed that treatment with the potent, non-toxic, and broad-spectrum caspase inhibitor Q-VD-OPh did not promote cortical neurogenesis, abolished ischemia-induced increase in proliferation, impaired ischemia-induced neural progenitor migration, and resulted in reduced levels of pro-inflammatory, but not anti-inflammatory, cyto/chemokines. The generation of cortical neurons after ischemia is controversial in animals and humans [2, 5, 6, 21]. In the present study, we showed that cortical neurogenesis is extremely rare, and most probably has no functional significance, at least not in the current experimental context. Neuronal progenitors have been shown to have phagocytic activity within the neurogenic niches under physiological conditions [22]. Moreover, NSPCs can regulate microglial function and activity [23]. The finding that neural progenitors continuously migrate to the injured cortex without turning into mature neurons, suggests that spontaneous replacement of cortical neurons after injury is likely not the biological mechanism. Therefore, the functional consequences of this migration process demand further elucidation, and caspase inhibitors constitute a useful tool in this quest.

Caspases are involved in other biological mechanisms besides cell death, for example inflammation. Caspase-1 is important for maturation of IL-1β and IL-18 protein and initiation of the inflammatory process [24]. Caspase-3, -7 and -8 are also important for microglia activation and neurotoxicity [25]. Our findings demonstrate that Q-VD-OPh appears to selectively interrupt the pro-inflammatory response after brain ischemia (Figure 4A-4D), however which particular caspase (s) involved in this context requires additional studies.

In a recent report, NSPCs committed to an astroglial phenotype were shown to migrate from the SVZ and contribute to the formation of a glial scar after cortical stroke [26]. In this study, we did not assess at the astroglial reaction, or other cell types that might be involved in the inflammatory reaction beside microglia, such as endothelial cells or blood-born cells, and whether Q-VD-OPh influences these cell populations is still unclear and requires further investigation.

In conclusion, despite the beneficial neuroprotective effects of the caspase inhibitor Q-VD-OPh in models of neonatal hypoxia-ischemia [27] and stroke [28], and the beneficial reduction of pro-inflammatory factors [29], it apparently also interferes with the endogenous NSPC regenerative response, and this should be taken into account when caspase inhibitors are included in neuroprotective strategies.

## MATERIALS AND METHODS

### Animals

Eight-week-old female C57BL/6 mice (Charles River, Sulzfeld, Germany) were used throughout the experiment. Animals were accommodated in equal light/dark cycles (12/12 hours) and given free access to food and water. All experimental procedures were performed in accordance with the European and Swedish animal welfare regulations and approved by Gothenburg ethical committee (application no. 290/09).

### Induction of cortical ischemia

Cortical ischemia was induced in the left hemisphere using a photothrombotic stroke model as previously described [5]. In brief, animals were anesthetized with isoflurane (5% induction and 1.5% maintenance; Isobavet; Schering-Plough Corporation, Kenilworth, NJ) in a mixture of air and oxygen (1:1). The body temperature was maintained with a heating pad during the experiment. An incision was made and the skin flaps were retracted to expose the skull. The photosensitive dye rose bengal (0.1 ml of 10 mg/ml) was injected intraperitoneally (IP) 5 min prior to laser illumination. The skull was illuminated for 10 min at the following coordinates relative to bregma: +1 mm anterior and +2.7 mm lateral. The laser beam settings were: power: 50 mW: wavelength: 561 nm; Cobolt Jive). The scalp was sutured and mice were kept in a heated box to recover. Sham control animals were anesthetized and injected with rose bengal, but the skull was not illuminated.

### Preparation and administration of the caspase inhibitor

The caspase inhibitor Q-VD-OPh was purchased from SM Biochemicals (Anaheim, California; cat. no. SMPH001). The inhibitor was initially dissolved in DMSO to generate a stock solution of 10 mg/ml. Aliquots were stored in −20°C. At the injection time, Q-VD-OPh aliquots were slowly diluted in 1x PBS (pH 7.4; Gibco/Life Technologies; San Diego, CA) to bring the DMSO concentration to 10%, then the injection solution was heated at 60°C for 20 min. Naïve controls, sham, or ischemic animals received IP injections of either 10 mg/kg Q-VD-OPh, a dose known to produce caspase-3 inhibition and tissue protection after hypoxia-ischemia [27, 29], or an equal volume of vehicle (10% DMSO) for 10-14 days, single daily injections (Figures 1A, 2A, and 3A ).

### Administration of 5-Bromo-2′Deoxyuridine

To assess proliferation in unlesioned animals (naïve controls), 2 IP injections of 5-Bromo-2′-Deoxyuridine (BrdU; 50 mg/kg; SigmaAldrich) were injected during the last 2 days of Q-VD-OPh treatment (Figure 1A). To birthdate the newborn cortical neurons in the ischemic animals, a single BrdU (50 mg/kg) injection was administered daily for 4 days, 8 weeks after induction of stroke (12 days prior administration of Q-VD-OPh) (Figure 2A).

### Tissue preparation

Animals were deeply anesthetized with sodium pentobarbital, transcardially perfused with 0.9% sodium chloride, and then fixed with 4% paraformaldehyde in 0.1M phosphate buffer (pH 7.4). Brains were collected and postfixed in the same fixative for 24 h, then transferred to 30% sucrose in 0.1 M phosphate buffer for cryoprotection, and left for a minimum of 3 days. Brains were cryosectioned coronally using a sliding microtome (Leica SM2000R) into 25 μm free floating sections and stored as 1:12 series at 4°C in tubes containing cryoprotection solution (25% glycerin, 25% ethylene glycol in 0.1M phosphate buffer) for further histological analysis.

### Immunohistochemistry and immunofluorescence

Sections were incubated in sodium citrate pH 6.0 for 30 min at 80°C when antigen retrieval was needed. For BrdU staining, sections were incubated in 2N HCl at 37°C for 30 min and neutralized with 0.1 M borate buffer for 10 min at room temperature. When the immunoperoxidase method was used, the endogenous peroxidase was quenched by incubating sections in 0.6% H_2_O_2_ for 30 min at room temperature. Non-specific binding was blocked by incubating sections in a solution of 3% normal donkey serum (Jackson ImmunoResearch Laboratories, West Grove, PA), 0.1% Triton X-100 in TBS for 1 h at room temperature. Sections were incubated at 4°C for 24-72 h with the primary antibodies. The following primary antibodies were used: rat anti BrdU (1:500, Serotec #OBT0030G); goat anti doublecortin (DCX; 1:200; Santa Cruz #sc-8066); mouse anti NeuN (1:200; Millipore #MAB377); rabbit anti Iba-1 (1:1,000; Wako #019-19741); rat anti mouse CD68 (1:500; Serotec #MCA1957T); rabbit anti Phospho-histone H3 (PHH3; 1:1,000; Millipore #06-570), followed by 1h or 2h incubation with appropriate biotinylated or fluorescent secondary antibodies, respectively. The following secondary antibodies were used: biotinylated donkey anti rat IgG; biotinylated donkey anti goat IgG; biotinylated donkey anti rabbit IgG (1:1,000; all from Jackson ImmunoResearch); Alexa-488 donkey anti goat IgG; Alexa-488 donkey anti rat IgG; Alexa-555 donkey anti mouse IgG; Alexa-555 donkey anti rabbit IgG (1:1,000; all from Molecular probes/Life technologies). ToPro3 (1:1,000; Molecular Probes/Life Technologies) was used as nuclear counterstain when fluorescence staining was used. To visualize the immunoperoxidase staining, sections were incubated for 1 h in avidin-biotin solution (1:100; Vectastain ABC Elite kit, Vector Laboratories, Burlingame, CA). The color precipitate was developed with H_2_O_2_, nickel chloride and 3-3′diaminobenzidine tetrahydrochloride (DAB; 1:100; Saveen Werner AB, Malmö, Sweden). Sections were mounted onto glass slides and coverslipped using Xtra-kitt mounting medium (Medite GmbH; Burgdorf; Germany) for immunoperoxidase staining or ProLong Gold anti-fade with DAPI (Molecular probes/Life technologies) for the fluorescent staining.

### Histological quantification

Analysis of BrdU and PHH3 immunoreactive cells in the SVZ was performed in a 1:12 series section interval covering the area +1.0 mm to −0.15mm anterior/posterior to bregma.

Quantification of migrating neural progenitors (doublecortin-positive cells; DCX+) and microglia (Iba-1-positive cells; Iba-1+) in the peri-infarct cortex was performed in 6 serial sections spaced 150 μm apart or 12 serial sections spaced 300 μm apart, respectively. All sections containing lesioned tissue were included. The peri-infarct area was delineated from the most central ventral part of the lesion and across the medial wall of the infarct tissue. A peri-infarct area of 300 μm (for DCX) or 100 μm (for Iba-1) in the surrounding intact cortex was included (Supplementary Figure 1). Quantification was based on counting only the cell soma of DCX+ and Iba-1+ cells.

The histological analysis was performed using a 40x objective lens in a microscope equipped with the stereology software Stereo Investigator (MicroBrightField Inc.) The total number of cells was the sum of all counted cells multiplied by the series interval.

### Immunofluorescence analysis and confocal microscopy

Activation of microglia was assessed by expression of CD68 (Iba-1+/CD68+) using an ApoTome microscope (AxioImager M2; Carl Zeiss microscopy, Germany). A minimum of 100 Iba-1+ cells were analyzed in image stacks with 0.5 μm optical slices taken through a 20x objective lens.

Quantification of DCX+ cells in the SVZ and the RMS, and colocalization of BrdU and NeuN (BrdU+/NeuN+) in the peri-infarct cortex were analyzed using a confocal laser scanning microscope (TCS SP2; Leica, Wetzlar, Germany). For DCX, analysis was performed in 12 serial sections spaced 300 μm apart covering the area +2.4 mm to −0.15 mm anterior/posterior to bregma. Image stacks were acquired with a 63x objective lens, and cells were considered for counting when the cell body of the DCX+ cell colocalized with the nuclear stain ToPro3. For BrdU+/NeuN+ analysis, image stacks were acquired with a 20x objective lens with 3x digital zoom. Confocal analysis was achieved using the following settings: optical slices of 1 μm or less; pinhole: airy 1.0; sequential fluorochrome excitation at 488 nm, 546 nm, and 633 nm.

### Tissue loss measurement

Lesioned sections 150 μm apart were mounted on Super Frost Plus glass slides, and left to dry overnight. Nissl staining was performed and areas were traced using the stereo investigator software (MicroBrightField Inc.). The percentage of tissue loss (%L) was determined by subtracting the area (A) of the intact tissue in the ipsilateral side (I) from area of the contralateral side (C) divided by the area of the contralateral side %L = [(Ac-Ai)/Ac] × 100.

### Protein extraction and ELISA

Animals were deeply anesthetized with isoflurane and decapitated. Brains were removed and hemispheres were separated. Lesioned tissue and surrounding cortex were carefully dissected and placed in a precooled 1.5 ml Eppendorf tube on dry ice. Ice-cold extraction buffer consisting of 50 mM Tris HCl (pH 7.4), 100 mM NaCl, 5 mM EDTA, 1 mM EGTA (all from Sigma-Aldrich), and protease inhibitor cocktail (Completemini; Roche; according to the manufacturer instructions) was added, and tissue was homogenized by sonication. Homogenates were centrifuged at 10,000 × g at 4°C for 10 min and the supernatants were collected. Protein concentration was determined using the BCA protein assay kit (Thermo Fisher Scientific Inc.; Rockford, IL), and samples were aliquoted and stored at −20°C. The concentrations of the cytokines interleukin-1 beta (IL-1β), interleukin-10 (IL-10), and the chemokines chemokine (C-C motif) ligand 2 (CCL2, also called monocyte chemotactic protein 1, MCP-1), stromal cell-derived factor 1 alpha (SDF-1α, also called C-X-C motif chemokine 12, CXCL 12), were measured using Quantikine^®^ Elisa kits (R&D systems Europe Ltd; Abingdon, UK). Samples were assayed undiluted in duplicates, and the assay was run according to the manufacturer's instructions.

### Statistical analysis

Data are presented as mean ± SEM. Student's *t*-test was used when comparing 2 groups. One-way ANOVA with Tukey (for histology) or Newman-Keuls (for cytokines) *post-hoc* test was performed when comparing more than two groups. Significance was considered when *P* < 0.05.

## SUPPLEMENTARY MATERIAL FIGURE


