# Supplementary material for: Caspase inhibition impaired the neural stem/progenitor cell response after cortical ischemia in mice
Source: Oncotarget. 2015 Dec 30;7(3):2239–48. doi: 10.18632/oncotarget.6803 (PMC4823032; doi:10.18632/oncotarget.6803)
Supplement: Supplementary file 1 [file oncotarget-07-2239-s001.pdf]

Caspase inhibition impaired the neural stem/progenitor cell response after cortical ischemia in mice

Supplementary Material

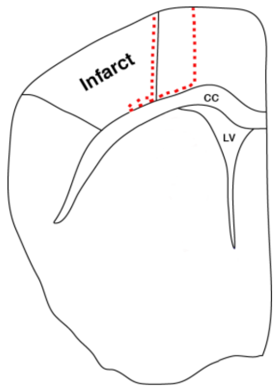

Supplementary figure 1

The dotted area displays the area of histological analysis.
